# Key Role of Obesity in Genitourinary Tumors with Emphasis on Urothelial and Prostate Cancers

**DOI:** 10.3390/cancers11091225

**Published:** 2019-08-22

**Authors:** Matteo Santoni, Alessia Cimadamore, Francesco Massari, Francesco Piva, Gaetano Aurilio, Angelo Martignetti, Marina Scarpelli, Vincenzo Di Nunno, Lidia Gatto, Nicola Battelli, Liang Cheng, Antonio Lopez-Beltran, Rodolfo Montironi

**Affiliations:** 1 Oncology Unit, Macerata Hospital, 62100 Macerata, Italy; 2 Section of Pathological Anatomy, School of Medicine, Polytechnic University of the Marche Region, United Hospitals, 60126 Ancona, Italy; 3 Division of Oncology, S.Orsola-Malpighi Hospital, 40138 Bologna, Italy; 4 Department of Specialistic Clinical and Odontostomatological Sciences, Polytechnic University of Marche, 60126 Ancona, Italy; 5 Medical Division of Urogenital and Head & Neck Cancer, IEO European Institute of Oncology IRCCS, 20141 Milan, Italy; 6 Dipartimento Oncologico USL Sud-Est Toscana-Area Senese, 53036 Poggibonsi, Italy; 7 Department of Pathology and Laboratory Medicine, School of Medicine, Indiana University, Indianapolis, IN 46202, USA; 8 Department of Pathology and Surgery, Faculty of Medicine, Cordoba University Medical School, 14004 Cordoba, Spain

**Keywords:** obesity, adipose tissue, urothelial cancer, prostate cancer, BMI, response to therapy

## Abstract

*Background*: In human populations, a certain amount of data correlate obesity/body mass index (BMI) with urothelial cancer (UC) and prostate cancer (PCa) occurrence, however this is not fully elucidated at all stages of disease. In an attempt to shed light on uncertain areas in such field, in the present review we illustrate the main molecular mechanisms linking obesity and cancer, focusing on the correlation between obesity and tumor risk, disease progression and response to chemo- and immunotherapy in patients with UC and the predictive/prognostic role of obesity in PCa patients treated with the currently available therapeutic approaches. *Methods*: We did a large-scale literature search on existing scientific websites focusing on keywords “obesity”, “body mass index (BMI)”, “urothelial cancer”, “prostate cancer”, “docetaxel”, “cabazitaxel”, “abiraterone acetate”, “enzalutamide”, and “radium223”. *Results*: Many adipocytes-induced molecules support tumor proliferation through activation of various cellular pathways. The available evidence in the postoperative setting do the role of BMI in oncological outcomes prediction still not completely clear. Likewise, in metastatic UC patients controversial results link the role of obesity/BMI with clinical outcomes of tumor response to chemotherapy. Adipose stromal cells recruitment, induced by PCa cells, from white adipose tissue to the tumor sites inducing cell invasiveness was associated with poor survival. Conflicting data, although more oriented towards a better survival outcome, resulted in obese patients treated with docetaxel. In PCa cell-lines a certain cabazitaxel chemo resistance adipose stromal cells (ASC)-mediated was demonstrated. In metastatic castration-resistant PCa patients with high BMI (>25 kg/m^2^) receiving abiraterone acetate there were significant worse survival outcomes, while in enzalutamide patients BMI did not affect survival outcome. In radium 223 patients higher BMI significantly correlated with favorable overall survival. *Conclusions*: The main focus of this review was to understand the interplay between obesity/BMI and UC/PCa. Several pathogenic cellular pathways exploring the issue are discussed, opening the way to challenging tailored treatments on the basis of BMI. Improving the knowledge of molecular connections between obesity and UC and PCa could favor the development of new therapies likely reducing chemo- and immunotherapy drug resistance.

## 1. Introduction

Obesity is a widespread disease defined as a fat tissue accumulation in body, rated as over 20% and 25% of total body weight in men and women, respectively [1]. This condition is also defined by a Body Mass Index (BMI) greater than 30 and is strictly associated to increased risk of diabetes, heart/cardiovascular disease and cancer [2]. Indeed, after the smoking habit, obesity is the second cause of carcinogenesis, and a certain amount of studies underlines stringent association among obesity and frequent cancers, such as gastrointestinal [3,4], gynecological [4], lung [5] and urinary tract cancers, including urothelial cancer (UC) and prostate cancer (PCa) [1,4]. Nevertheless, which biological mechanisms correlate obesity with an increased risk of cancer development is still unclear. Indeed, adipocytes-derived molecules are able to modulate the activity of several pathways, including PI3K/AKT, mammalian Target of Rapamycin (mTOR)/cyclin D1, mTOR/HIF1A/Vascular Endothelial Growth Factor (VEGF) and Ras, leading to inhibited apoptosis, stimulated cell proliferation and neoangiogenesis [6,7,8,9,10]. In this review we review the main mechanisms linking obesity and cancer, focusing on the correlation between obesity and tumor risk, progression and response to therapy in patients with metastatic UC and prostate cancer.

## 2. Molecular Mechanisms Linking Obesity and Cancer

**Figure 1:**
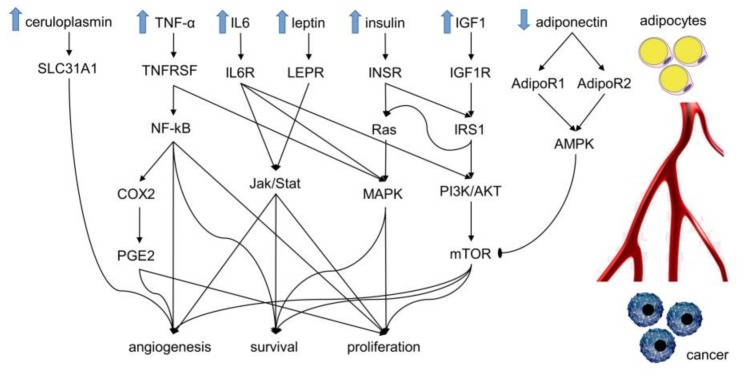
The pathway shows the influence of adipocytes on cancer. The arrows indicate induction or activation, the line ending with an oval segment (AMPK on mTOR) indicates inhibition. The substances released from adipocytes diffuse through the blood circulation and reach cancer cells where activate different cellular pathways. The latter lead to an increase of cell proliferation, survival and angiogenesis. IGF1: Insulin-like growth factor I. IGF1R: Insulin-like growth factor 1 receptor. INSR: Insulin receptor. IRS1: Insulin receptor substrate 1. BAD: Bcl2-associated agonist of cell death. MAPK: Mitogen-activated protein kinase. LEPR: Leptin receptor. ADIPOR1: Adiponectin receptor protein 1. ADIPOR2: Adiponectin receptor protein 2. VEGFA: Vascular endothelial growth factor A. AMPK: AMP-activated protein kinase. mTOR: Serine/threonine-protein kinase mTOR. CP: Ceruloplasmin. SLC31A1: High affinity copper uptake protein 1. TNF: Tumor necrosis factor. TNFRSF: Tumor necrosis factor receptor superfamily. IL6: Interleukin-6. IL6R: Interleukin-6 receptor subunit alpha. PTGS2: Prostaglandin G/H synthase 2. PI3K: Phosphoinositide 3-kinases. AKT: serine/threonine kinase. HIF1A: Hypoxia-inducible factor 1-alpha.

In this section we describe the molecular mechanisms that show the association between obesity and cancer. Figure 1 shows the main molecules produced by adipocytes and how support tumor proliferation by activating various cellular pathways. In particular, obese individuals have insulin resistance with high circulating insulin and insulin-like growth factor I (IGF1) levels. This is often associated with activation and/or up-regulation of insulin-like growth factor 1 (IGF1R) and insulin (INSR) receptors that, by interacting with insulin receptor substrate 1 (IRS1), activate PI3K/AKT, mTOR/cyclin D1, mTOR/HIF1A/VEGF and Ras pathways. These axes inhibit apoptosis, stimulate cell proliferation and angiogenesis [7,10,11,12]. Moreover, insulin resistance leads to higher blood glucose level which might be also an important factor for cancer growth. Indeed, cancer cells prefer to use predominantly the glycolysis (Warburg effect) to produce their energy, even in aerobic conditions [13].

Obesity is also associated with increased levels of blood fatty acids. Fatty acids and in particular, polyunsaturated fatty acids (PUFAs) affect cancer risk and progression. Tumor cells tend to increase the production of fatty acids by the upregulation of fatty acid synthesis-related enzymes. This increased activity is achieved through signalling by mTOR and sterol regulatory element-binding proteins (SREBPs), which stimulate proliferation in cancer cells through Akt signalling [14,15,16,17].

Leptin is a hormone produced from adipocytes and present at high level in obese individuals. It activates and up-regulates its receptor (LEPR) that, in turn, promotes cell proliferation, survival and angiogenesis through Jak/Stat, PI3K/AKT and MAPK pathways. Adiponectin, by its receptors ADIPOR1 and ADIPOR2, blocks pro-cancer effects of mTOR and, in particular, prevents angiogenesis [18].

Unfortunately, contrary to other adipose-derived factors, adiponectin is present at low levels in obese individuals. Ceruloplasmin is an adipose-derived factor that binds copper, which influences the VEGF production through SLC31A1 receptor [19]. High levels of ceruloplasmin in obese individuals promotes angiogenesis and the development of cancer. Adipocytes produce pro-inflammatory cytokines, such as TNF-α and IL6, that induce pro-tumorigenic cyclooxygenase 2 (COX2 or PTGS2) expression, causing the production of prostaglandin E2 (PGE2) that promotes tumor progression.

In addition, PGE2 can induce tumor epithelial cells to secrete growth, pro-inflammatory and angiogenic factors, and shift the tumor microenvironment to an immunosuppressive response [20]. Instead, NF-kB TNF-α activated, is a transcription factor that promotes cell survival through the up-regulation of anti-apoptotic factors (BCL-2, survivin, etc.), cell proliferation through up-regulation of cyclin D1 and cyclin E, inflammation through up-regulation of cytokines (IL-1, IL-2, IL-6, etc.), and angiogenesis. Finally, interleukin-6 (IL6), through its receptor (IL6R), enhances Jak/Stat and PI3K/AKT pathways resulting in anti-apoptotic and proliferative effects.

## 3. Obesity and Risk of Urothelial Cancer

About 80,000 new cases of UC of the urinary bladder and urinary systems are estimated in 2019 [21]. UC is a heterogeneous disease characterized by metabolic alterations that lead to an increased expression of genes that favor the pentose phosphate pathway (glucose-6-phosphate dehydrogenase (G6PD)) and the fatty-acid synthesis (fatty acid synthase (FASN)), together with decreased AMP-activated protein kinase (AMPK) and Krebs cycle activities [9].

**Figure 2:**
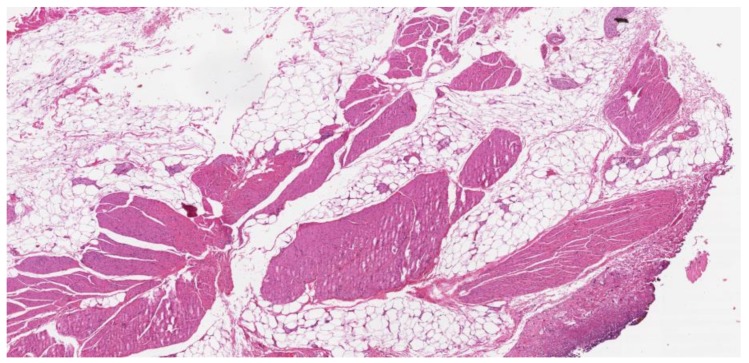
Abundant adipose tissue within the bladder wall, partially replacing the muscularis propria. 20× magnification.

Thanks to the availability of several therapeutic approaches in both early and advanced settings, the survival of patients with UC has been significantly improved in the last years. One of the recognized risk factors for bladder cancer (BC) development is obesity. Adipocytes can be abundant in tumor microenvironment (Figure 2), and their role in this setting appears of particular interest. Of note, other than an increased risk of disease development lifetime, the presence of obesity and more generally the correlation between BMI and clinical outcome in the different stages of the disease result fundamental.

Endoscopic disease exportation with/without local instillation of chemotherapy or Calmette-Guérin Bacille (BCG) still remain the standard approaches for non-muscle invasive urothelial bladder tumors. In muscle invasive UC, the only curative approach is represented by radical surgery, which consists of a radical cystectomy (RC) or radical nephroureterectomy (RNU) in BC and upper tract UC (UTUC) respectively [22].

In patients who undergo to radical surgery, evaluation of body BMI is one of the most evaluated and discussed parameter able to estimate and predict clinical outcomes including cancer specific survival after surgery. Several mono and multi centers studies have evaluated the correlation between BMI index and oncological outcomes. The largest retrospective series was carried out on 4118 patients treated with RC and pelvic lymphadenectomy (without preoperative chemotherapy/radiotherapy). This retrospective analysis identified BMI as a strong predictor of worse cancer-specific outcomes. In particular, obesity was strongly associated to higher risk of disease recurrence, cancer and overall mortality [6]. Similarly, other retrospective studies confirmed the prognostic value of higher BMI in patients with both BC or UTUC [23,24] while another series suggested that also lowest value of BMI was associated to worst oncological outcomes [25]. On the contrary, other retrospective studies failed to find an association between BMI and oncological outcomes after surgery [26,27] while other confirmed a prognostic role only after RC without significant association after RNU [28,29]. Due to the absence of external and perspective validation of the results observed in retrospective series, the role of BMI in oncological outcomes prediction after surgery still remains unclear.

## 4. BMI and Response of Urothelial Cancer to Chemotherapy and Immunotherapy

Another issue to consider is that patients who undergo to perioperative chemotherapy have been usually excluded from these series. As known, platinum-based chemotherapy represents the standard treatment for patients with muscle invasive bladder cancer while few evidences are available for UTUC [30,31,32]. Very few data are available about the correlation between BMI and clinical outcomes after neoadjuvant chemotherapy. In a small cohort of patients with muscle-invasive urothelial carcinoma of the bladder treated in a single institution, the administration of platinum based chemotherapy resulted in a significant decrease in lean muscle mass and higher prevalence of sarcopenia [33].

Metastatic UC is a disease associated to poor prognosis with an expected 5 years-overall survival (OS) of only 4–5% [21]. In this setting, cisplatin combination regimens are the standard first line therapies while patients unfit to cisplatin are generally treated with carboplatin regimens or platinum-free combinations [34,35,36,37]. Until few years ago, there were very few options for patients progressed to first line treatment and vinfluine was the only treatment which was associated to OS improvement over best supportive care [38]. In Europe, vinflunine has become the standard second-line therapy for patients progressed on first-line or perioperative platinum-containing regimen [39]. Alternative regimens based on the administration of taxans and/or gemcitabine can be valuated case by case [40,41].

Immune-checkpoint inhibitors have represented a revolution for the management of metastatic UC. These are results of the fundamental advances in understanding the role of inflammatory mediators in UC carcinogenesis [42] and of the discovery of a series of promising immunotargets [43] that have led to the development of agents targeting the Programmed Death Protein 1 (PD-1) or the Programmed Death Protein Ligand 1 (PD-L1). These agents are able to restore immune response against tumor. Pembrolizumab was the first agent approved by FDA for patients progressed to standard first line therapy [44]. To date other immune-agents have showed to improve clinical outcomes of patients with metastatic UC and evaluation of these agents in other setting of the disease in under evaluation [45,46,47]. In metastatic patients the role of BMI seems to be associated to prognosis and oncological outcomes. In particular, sarcopenia seems to be a parameter related to shorter survival. Two single-institution retrospective analyses showed that reduction of skeletal muscle masses is related to poorer clinical outcomes. In a Japanese cohort of 88 patients with advanced (un-operable) or metastatic UC skeletal muscle index was measured from computed tomography (CT) at the diagnosis and stratified for BMI. In this small series, the presence of sarcopenia was significantly related to poor prognosis (Hazard Ratio = 3.36; *p* > 0.001) with a median survival of 11 and 31 months in patients with and without sarcopenia [48]. Similar result has been obtained in a subsequent retrospective study carried out on 87 metastatic UC patients who underwent to first line therapy with cisplatin-gemcitabine or carboplatin-gemcitabine chemotherapy. Also this study confirmed that skeletal muscle index stratified for BMI resulted a strong predictor of survival in this population (Hazard Ratio = 3.102; *p* = 0.026) [49]. A large study carried out on 537 patients enrolled in eight phase II and phase III clinical trials and receiving cisplatin based combination for metastatic UC investigated the role of obesity on clinical outcomes. In this large population, the presence of obesity did not correlate with number of chemotherapy cycles, adverse events, response rate, progression free survival and overall survival. Only embolic events and renal failure were more frequent in patients with higher BMI [50].

No studies have evaluated the correlation between immune-checkpoint inhibitors outcomes and BMI in patients with UC. Of interest very recently a large multicenter study evaluated this issue among patients with lung cancer, melanoma, kidney cancer and other diseases. It is very interesting to observe that among the 976 patients receiving immune-checkpoint inhibitors, overweight/obese patients showed higher response rate, longer time to treatment failure, progression free survival and overall survival [51]. Authors concluded that overweight/obesity could be considered as a tumorigenic immune-dysfunction and that immune-checkpoint inhibitors could reverse this condition leading to improved clinical outcomes. Future studies aimed to assess this issue on a cohort of patients with metastatic UC are needed (Table 1).

## 5. Key Issues on Obesity and Urothelial Cancer

The outcome of patients with advanced UC has definitively improved in the last decade due to the introduction of novel chemotherapies [38] and immunotherapies [44,45,46]. Nevertheless, the rate of patients with complete responses to therapy is still poor. This may be partially explained by the complex molecular background of this disease, which makes difficult the identification of key driving alterations. Epithelial Growth Factor Receptor (EGFR), Fibroblast Growth Factor Receptor (FGFR), VEGFR, PI3K/AKT/mTOR pathway, PD-1, COX2, Aurora kinase A and miRNA are just examples of this new frontier [52]. Interestingly, some of these axes, in particular PI3K/AKT/mTOR, result activated in obese patients due to the high circulating levels of IGF1, thus representing a potential therapeutic target in UC patients with high BMI.

Personalizing cancer therapy for UC patients is currently one of the major goals for uro-oncologists worldwide [53]. The possibility to personalize treatments on the basis of BMI represents an interesting challenge, but only through the comprehension of the alterations in tumor microenvironment of obese UC patients we will be able to tailor future therapeutic strategies. Indeed, UC patients treated with either chemotherapy or immunotherapy do develop acquired resistance to these approaches [54,55]. Interestingly, it has been showed that the expression of Nuclear factor erythroid 2–related factor2 (Nrf2), a leucine zipper transcription factor regulating cellular detoxification and antioxidant response through the induction of the expression of heme oxygenase 1 (HO-1), GST, NAD(P)H-quinone oxidoreductase (NQO1), glucuronosyltransferase-1a6 (UGT-1a6), and superoxide dismutase 3 (SOD3) [56], is correlated with resistance to cisplatin. Moreover, the expression of Nrf2 has been associated with the Recurrence-Free Survival (RFS) of BC patients who undergo neoadjuvant chemotherapy followed by cystectomy [57]. Of note, Nrf2 is implicated in adipocyte differentiation, adipogenesis, obesity, and insulin resistance [58,59].

Another alteration associated with response to therapy is represented by the presence of *BRCA* mutations, in particular *BRCA1*. Indeed, it has been shown that BC patients with low/intermediate mRNA levels of BRCA1 receiving neoadjuvant platinum-based therapies presented increased tumor pathological response and OS in comparison with patients with high mRNA BRCA1 levels [60]. The connection between BRCA1 mutations and obesity has been investigated, showing that overweight and weight gain increased postmenopausal breast cancer risk in BRCA1/2 mutation carriers [61]. On this scenario, the relationship between obesity and BRCA1 mutations in UC patients should be further explored, even due to the necessity of understanding the potential role of Poly (ADP-ribose) polymerase (PARP) inhibitors in this tumor. At this regards, a phase II clinical trial is in course to assess the efficacy and safety of PARP inhibitor olaparib in patients with advanced or metastatic UC and DNA-Repair defects progressed during at least one platinum-based regimen of chemotherapy and/or an immune-checkpoint inhibitor (NCT03375307).

## 6. Obesity and Prostate Cancer

Prostate cancer (PCa) is one of the leading causes of cancer-related death worldwide, accounting for 180,890 new cases and over 26,000 deaths in the United States in 2016 [62]. The last two decades have been characterized by fundamental improvements of our knowledge on the complexity of prostate carcinogenesis [63,64,65]. This process is the result of a series of events that involves: (1) wide simultaneous genomic rearrangements leading to double-strand DNA breaks (a phenomenon known as “chromoplexy”) [66]; (2) metabolic alterations (including over-expression of major lipogenic enzymes such as FASN and HMG-CoA reductase) and deregulation of the energy sensor 5-AMP-activated protein kinase, AMPK) [67]; (3) immune cells, in particular neutrophils and tumor associated macrophages (TAMs) [68,69,70], which contribute to the creation of a favorable microenvironment for PCa growth and invasion; (4) neuroendocrine cells, defined by the presence in the cytoplasm of markers such as chromogranin A (CgA) and neuron-specific enolase (NSE), implicated in PCa aggressiveness [71,72]; (5) changes in human urinary microbiota [73], which may result relevant in PCa carcinogenesis and response to therapy.

**Figure 3:**
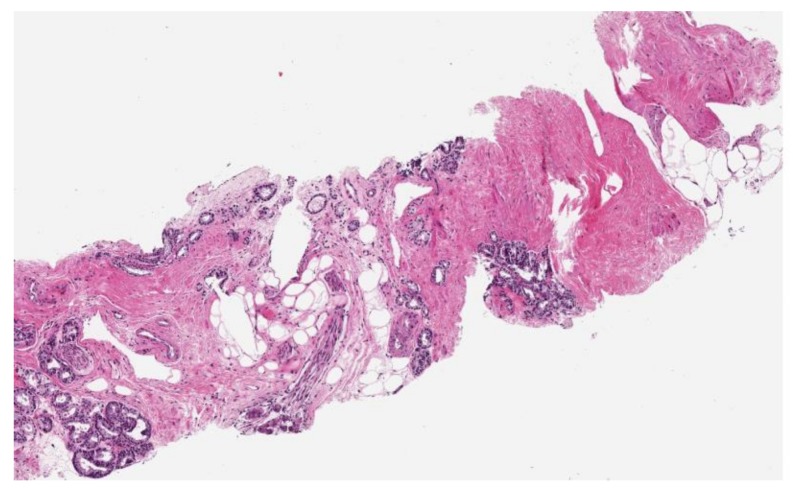
Prostate biopsy showing prostate acinar adenocarcinoma (Gleason score 3 + 4 = 7, grade group 2; percentage of Gleason pattern 4 equal to 40%; Gleason pattern 4 architecture fused glands). The tumor has spread to the periprostatic soft tissue composed by adipose and fibrous tissue and nerves. Fibrosis basically replaced the adipose tissue when infiltrated by cancer, i.e., stromal reaction. 10× magnification.

The improved possibility to characterize tumor histology (Figure 3) and molecular background [74,75] and, as a consequence, patients’ prognosis [76] has led to an increase in patients’ outcome. However, further steps forward are needed in the management of this disease.

An emerging topic that may be the key to optimize the prevention, treatment and follow-up of this tumor is represented by life style. Numerous epidemiological studies have examined the occurrence of PCa in obese patients and hence several pathogenic mechanisms have been proposed, including chronic inflammation [77,78]. Furthermore, obesity has been recognized as a key prognostic factor in patients receiving either chemotherapy or hormone therapy for advanced PCa [79].

## 7. Obesity and Risk of Prostate Cancer

Chronic inflammation is thought to create a link between obesity and PCa. Indeed, inflammation is a usual finding in prostate samples and the major causes may enclose bacteria-induced prostatitis, the influence of high estrogen levels, physical trauma, urine reflux toward prostate, and dietary costumes [80,81]. Certain published data underline that the obesity induces a systemic inflammation across various proposed mechanisms, here briefly described as follows. In obese men, adipocytes secrete pro-inflammatory cytokines, such as tumor necrosis factor-α (TNF-α), interleukin-1 (IL-1) and IL-6 that induce chronic inflammation [77]. Along this line, macrophages in fat tissue appear enhanced in obese patients and exhibit a pro-inflammatory habit contributing to TNF-α production [82,83]. On the same level, the occurrence in obese people of increased insulin level and insulin-like growth factor (IGF-1) in blood, phenomena known as insulin-resistance, may lead to tumor proliferation [84]. Such mechanisms ultimately would increase the risk of PCa occurrence. Intriguingly, certain cytokines and chemokines released from PCa cells would be able to recruit immune cells to the prostate. To substantiate this, high expression of macrophage inhibitory cytokine 1 (MIC1) has been found in obese patients with PCa [85]; a high-fat diet stimulates several C-X-C motif ligands, and in particular CXCL1, which promotes the recruitment of adipose stromal cells (ASC) from white adipose tissue (WAT) to the tumor, finally inducing tumor growth [86]; a very fat diet may also induce increased leptin level, a hormone hypothesized to support cancer cell proliferation [87]. These evidences would demonstrate the role of certain cytokines/chemokines behind the link between obesity and PCa development. Of note, in obese PCa people ASC trafficking from WAT to tumors has been associated with poor survival [88]. Both adipocytes and ASC secrete factors termed adipokines that may exhibit a tumor-trophic habit. Lengyel and colleagues have recently demonstrated that mitogenic adipokine signaling as well as anti-apoptotic and paracrine angiogenic signals strongly support the crosstalk between adipose cells and tumor development [89]. The role of ASC has been very well recently elucidated in cell culture and mouse models of PCa, in which ASC induced epithelial-mesenchymal transition, a phenomenon linked to stimulation of chemoresistance, cell invasiveness and metastatic dissemination [89].

## 8. Correlation between Obesity and Response to Therapy in Prostate Cancer

Taking into account these premises and moving into a practical scenario in patients with PCa, we are aware that the influence of overweight/obesity on clinical outcomes of specific therapies is poorly known. Accordingly, in the present article we aim to shed light on the relationship among overweight/obesity and the currently approved drugs (including docetaxel and cabazitaxel chemotherapeutic agents, new generation hormone therapy abiraterone acetate and enzalutamide and radioactive agent radium 223) in patients with metastatic PCa, here reported.

### 8.1. Docetaxel

Some data would suggest that obesity may influence the outcomes of PCa patients treated with docetaxel chemotherapy. In patients with metastatic castration-resistant PCa (mCRPC) recruited to the seminal phase III TAX327 study [90], Armstrong et al. retrospectively speculated the predictive role of baseline body mass index (BMI) comparing baseline features in obese (>30 kg/m^2^) versus non-obese patients (18–29 kg/m^2^). The authors did not find any significant correlation between obesity and PCa outcomes including survival data and PSA declines [91]. In a large retrospective cohort of metastatic PCa patients, Wu et al. interestingly observed that obese patients were treated with docetaxel earlier than patients with normal BMI (<25 kg/m^2^), however without any impact in survival, and that weekly docetaxel regimens and obesity were significant predictors of longer survival after docetaxel treatment [92]. Along this line, Cushen and colleagues performed a retrospective analysis of mCRPC patients receiving three-weekly or biweekly docetaxel provide that overweight/obese patients (BMI >25 kg/m^2^) had a better overall survival than patients with BMI <25 kg/m^2^ [93].

### 8.2. Cabazitaxel

In PCa cell lines, Su and co-workers have recently tested whether ASC do cancer cells more resistant to cabazitaxel chemotherapy. Cabazitaxel as expected induced a dose-dependent reduction in numbers of LNCaP and PC3 cell lines, whereas LNCaP cells incubated with ASC for 24 hours resulted still survived at cabazitaxel. Using in co-culture peptide D-CAN that is lethal toward ASC, cabazitaxel cytotoxicity was restored, thus demonstrating ASC-mediated chemoresistance to cabazitaxel [89].

### 8.3. Abiraterone Acetate and Enzalutamide

A few years ago, Cavo et al. evaluated the correlation between risk factors of adverse events and survival outcomes in mCRPC patients treated with abiraterone acetate and prednisone, observing that at multivariable analysis BMI >25 kg/m^2^ resulted significantly associated both with worse progression-free survival and worse overall survival (OS) (*p* = 0.03 and *p* = 0.042, respectively) [94]. Furthermore, a research group headed by Antoun retrospectively analyzed whether body composition parameters affected prognosis of mCRPC patients treated with androgen receptor inhibitors abiraterone acetate and enzalutamide into two prospective clinical trials [95,96], showing that no relationship between BMI and OS was detected [97].

### 8.4. Radium 223

The relationship between obesity and the use of Dichloride radioactive therapeutic agent Radium 223 has still not been clarified so far. At this regard, Frantellizzi et al. [98] collected a series of 92 mCRPC patients with symptomatic bone metastases who received Radium 223. In this study, with a median follow-up of 6 months, patients’ weight, BMI, Eastern Cooperative Oncology Group-Performance Status (ECOG-PS), Hemoglobin (Hb) and total alkaline phosphatase (tALP) were significantly correlated with OS at univariate analysis, while only ECOG-PS and Hb levels were significant predictors of OS at multivariate analysis (Table 2).

## 9. Key Issues on Obesity and Prostate Cancer

During the last decades, cancer has continued to represent a worldwide killer, despite the long series of advances in understanding tumor biology and novel therapeutic agents introduced into clinical practice. Among the millions of cases diagnosed worldwide, only 5–10% can be associated with gene alterations, while 90–95% can be correlated with environmental factors [99]. The relationship between lifestyle and risk of cancer has been fully investigated in the last years. The list of lifestyle factors includes smoking attitude, diet, alcohol consumption, obesity, environmental exposure, infections, stress, and physical inactivity [100].

Obesity does not only represent a risk factor for genitourinary tumors but also a prognostic factor. According to the American Cancer Society, obesity increases the risk of cancer-related mortality, representing together with overweight the main cause of death in cancer patients in 14% of men and 20% of women [4]. Indeed, targeting obesity does constitute a promising approach for cancer patients, although this possibility is still limited by the wide spectrum of signalling pathways involved in obesity-related cancer risk and response to therapies [101]. Only through the development of specific multi-target agents we could finally reduce the impact of obesity on life expectancy and optimize cancer prevention.

## 10. Conclusions

A better comprehension of the relationship between obesity and UC will optimize the outcome of these patients through the identification of novel therapeutic targets in obese patients. This will open the way to personalized therapy in obese UC patients and will allow to understand the role of adipocytes and, as a consequence, to reduce the development of drug resistance to both chemotherapy and immunotherapy. Advances in understanding the connection between obesity and PCa will improve patients’ outcomes and quality of life and will promote the development of new personalized strategies for both follow-up and treatment. Acting on lifestyle results fundamental to significantly reduce the carcinogenic and prognostic role of obesity worldwide.

## Figures and Tables

**Table 1:** BMI and response of urothelial cancer to chemotherapy and immunotherapy.

Patients	Results	Reference
88 mUC patients	SMI was a significant and independent predictor of shorter OS (HR 0.90, *p* < 0.001). Median OS rates were 11 and 31 months for sarcopenic and non-sarcopenic patients; sarcopenia was a significant and independent predictor of shorter OS (HR 3.36, *p* < 0.001)	Fukushima et al., 2015 [48]
87 mUC patients who underwent chemotherapy	SMI stratified by the value of the BMI was a significant predictor of shorter OS in univariate analysis (*p* = 0.037) HR = 3.102; *p* = 0.026	Abe et al., 2018 [49]
537 mUC patients treated with cisplatin-based combination therapy	Embolic events and renal failure were higher in patients with an average or higher BSA. Obese patients have similar response rates, survival outcomes, and tolerability of cisplatin-based therapy to non-obese patients.	Leiter et al., 2016 [50]
976 patients with different tumors: NSCLC (65.1%), melanoma (18.7%), RCC (13.8%) and others (2.4%)	Median TTF, PFS and OS were significantly longer for overweight/obese patients in univariate (*p* < 0.0001, for all the survival intervals) and multivariate models.	Cortellini et al., 2019 [51]

mUC: metastatic urothelial carcinoma; NSCLC: Non-small cell lung cancer; RCC: renal cell carcinoma; SMI: Skeletal muscle index; BMI: body mass index; BSA: body surface area; HR: Hazard ratio; TTF: time to treatment failure; PFS: progression free survival; OS: overall survival.

**Table 2:** Obesity and response to therapy in prostate cancer.

Drug	Patients/Cells	Results	Reference
Docetaxel	1006 CRPC patients	Obesity was associated with younger age, lower PSA and tALP, and higher performance status, primary Gleason sum, testosterone and Hb. In multivariate analysis, neither BMI, presence of obesity, nor baseline testosterone was significantly associated with OS or PSA declines.	Armstrong et al., 2009 [91]
	333 mCRPC patients	High VMR, obesity, and weekly regimens were significant predictors of longer survival after docetaxel.	Wu et al., 2015 [92]
	63 mCRPC patients	In multivariate analysis, BMI ≥25 kg/m^2^ (HR: 0.349, CI: 0.156–0.782, *p* = 0.010) was a significant predictor of longer OS and both visceral fat index ≥ median 58.7 cm^2^/m^2^ (HR: 2.266 CI: 1.066–4.814, *p* = 0.033) and anaemia (HR: 2.81, CI: 1.297–6.091, *p* = 0.009) were significant predictors of shorter OS.	Cushen et al., 2016 [93]
Cabazitaxel	Human cell co-culture models	ASC-mediated chemoresistance to cabazitaxel. ASC induce epithelial-mesenchymal transition in prostate cancer cells.	Su et al., 2019 [89]
Abiraterone acetate and Enzalutamide	105 patients	At multivariable analysis BMI >25 kg/m^2^ resulted significantly associated both with worse progression-free survival and worse OS (*p* = 0.03 and *p* = 0.042, respectively)	Cavo et al., 2018 [94]
	120 patients mCRPC	High volume of SAT is independently associated with OS.	Antoun et al., 2015 [97]
Radium223	92 mCRPC patients	Patients’ weight, BMI, ECOG-PS, Hb and tALP significantly correlated with OS at univariate analysis, while only ECOG-PS and Hb levels were significant predictors of OS at multivariate analysis.	Frantellizzi et al., 2018 [98]

mCRPC: metastatic prostate cancer; BMI: Body Mass Index; OS: overall survival; PFS: progression-free survival; VMR: visceral fat-to-muscle area ratio; ASC: adipose stromal cells; SAT: subcutaneous adipose tissue; ECOG-PS: Eastern Cooperative Oncology Group-Performance Status; Hb: Hemoglobin; tALP: total alkaline phosphatase.
